# A proteomic perspective and involvement of cytokines in SARS-CoV-2 infection

**DOI:** 10.1371/journal.pone.0279998

**Published:** 2023-01-06

**Authors:** Sarena Banu, Ramakrishnan Nagaraj, Mohammed M. Idris

**Affiliations:** CSIR-Centre for Cellular and Molecular Biology, Hyderabad, India; Rutgers Biomedical and Health Sciences, UNITED STATES

## Abstract

Infection with the SARS-CoV-2 virus results in manifestation of several clinical observations from asymptomatic to multi-organ failure. Biochemically, the serious effects are due to what is described as cytokine storm. The initial infection region for COVID-19 is the nasopharyngeal/oropharyngeal region which is the site where samples are taken to examine the presence of virus. We have now carried out detailed proteomic analysis of the nasopharyngeal/oropharyngeal swab samples collected from normal individuals and those tested positive for SARS-CoV-2, in India, during the early days of the pandemic in 2020, by RTPCR, involving high throughput quantitative proteomics analysis. Several proteins like annexins, cytokines and histones were found differentially regulated in the host human cells following SARS-CoV-2 infection. Genes for these proteins were also observed to be differentially regulated when their expression was analyzed. Majority of the cytokine proteins were found to be up regulated in the infected individuals. Cell to Cell signaling interaction, Immune cell trafficking and inflammatory response pathways were found associated with the differentially regulated proteins based on network pathway analysis.

## 1. Introduction

The COVID19 pandemic has led to extensive investigations on multiple aspects of the biology of severe acute respiratory syndrome coronavirus 2 (SARS-CoV-2) virus as well as host-responses [1, 2]. Despite extensive investigations, as of now, vaccination appears to be the only way to avoid or reduce infection by the virus [1, 2]. To date, testing for the virus relies on swabs from nasopharyngeal region followed by detection of the virus by Real time polymerase cycle reaction (RTPCR) [1–3]. The nasopharynx is the initial area of infection and is close to the lungs which are affected in severe cases of SARS-CoV-2 infection. Hence, it is important to investigate early events at the initial site of infection immediately after detection by RTPCR. The early events related to host-response at the site of infection, may give a clue about the disease progression and the protein regulation at an early stage of infection.

There have been detailed proteomics studies on the serum of SARS-CoV-2 patients were conducted using liquid chromatography with Tandem mass spectrometry (LC-MS/MS) [4–10]. The proteins from serum were digested with trypsin and the peptides were subjected to LC-MS analysis [4, 6, 8]. In two studies [5, 10], peptides obtained from digesting serum or cellular proteins were pre fractionated by LC, fractions pooled and the pooled peptides were subjected to LC-MS/MS analysis. D’Alessandro et al have examined the effect of IL-6 levels on coagulation and complement status using nano Ultra-High-Pressure Liquid Chromatography-Tandem Mass Spectrometry [4]. The study by Villar et al describes dysregulation of proteins on COVID-19 infection and have suggested that immune related markers would aid in monitoring the pandemic [7]. Park et al describe changes of plasma proteins and differences between severe and non-severe COVID-19 patients [8]. Shen et al have reported detailed proteomic and metabolic analysis from serum of patients with COVID19 using Ultra performance liquid chromatography/ Tandem Mass Spectrometry (UPLC-MS/MS). Their detailed study indicated massive metabolic suppression and molecular changes in blood induced by SARS-CoV-2 [5]. Messner et al have developed a high throughput platform for serum and plasm proteomic analysis developed for clinical use. Their study revealed differentially expressed proteins that may have potential as biomarkers. The proteins included complement factors and inflammation modulators [6]. However, only one study has been conducted to explain the immune response on the nasopharyngeal (NP) region [10]. Based on proteomic analysis of samples from the NP region [10], Vanderboom et al. explained that upon SARS-CoV-2 infection, the innate immune system activated interferon (IFN)-mediated proteins on the NP region, which includes interferon-stimulated genes, pathogen-recognition receptors, transcription factors, and proinflammatory cytokines [10]. Interestingly, only one common up-regulated protein was found between NP and serum samples. There was no down-regulated protein in common [10]. The functions of nasal-associated lymphoid tissue (NALT) and its immune cells such as B cells, T cells, macrophages, and dendritic cells on innate and adaptive immunity following SARS-CoV-2 infection have not been well described. The mucosal immune system is the largest; it mediates both innate and adaptive immunity against SARS-CoV-2 infection by circulating antibodies such as Secretory (SIgA), IgA and IgG via (Nasal-associated lymphoid tissue) NALT to neutralize viral infection [11]. Hence, it is relevant to examine the effect of virus attack on the cells of this region. We have shown that upon infection, there is down regulation of defensin genes [12]. Defensins are crucial components of innate immunity and are the first line of defense against invading pathogens [12, 13]. In the present study, we have now analyzed samples obtained during early days of the pandemic in 2020, for expression of protein profiles, particularly their regulation with respect to controls. We have also in parallel examined differential expression of different cytokines. We have observed up regulation of several proteins related to inflammation and innate immunity. Of particular interest is the up regulation of annexins. Auto antibodies to annexins have been observed in COVID19 patients [14]. The up regulated proteins may result in the production of auto antibodies which contributes to metabolic dysfunction observed in the disease. We have observed up regulation of several cytokines at the genomic and protein level. These cytokines play a role in inflammation. Our results indicate that metabolic stress due to viral infection is observed in nasopharyngeal cells at the onset of infection.

## 2. Results

The Nasopharyngeal region comprises of a heterogeneous population of cells that include epithelial and immune cells [11, 15]. The Nasopharyngeal region is also gateway to the lungs which is affected badly when the illness is serious. The detection of SARS-CoV-2 infection involves RTPCR from Nasopharyngeal/oropharyngeal swab (NOPS) samples. We have earlier shown that analysis of cells from this region indicates down regulation of defensin gene expression [12]. We describe in this paper, differential proteomics analysis and expression of some cytokine genes from NP swabs from control population and those infected with SARS-CoV-2 as indicated by RTPCR.

### 2.1. Proteomics analysis

**Fig 1:**
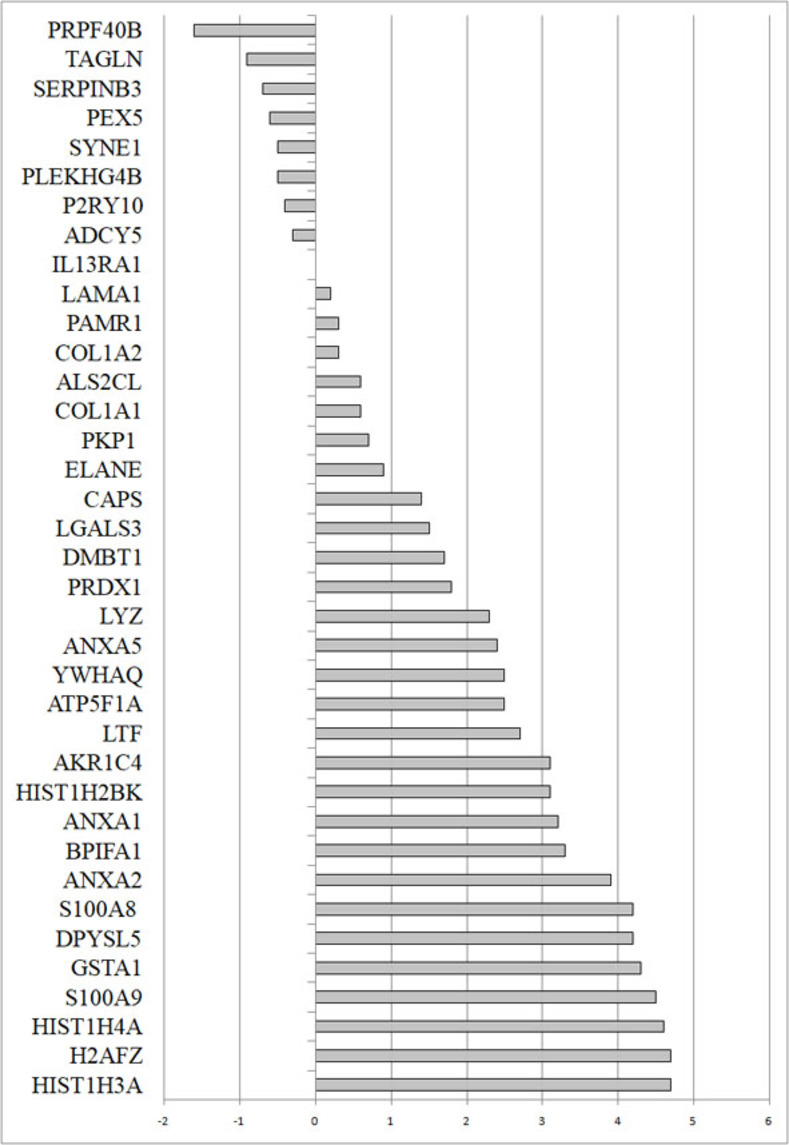
Proteins associated with SARS-CoV-2 infection. List of proteins associated with SARS-CoV-2 infected NOPS samples against control negative samples. The X-axis represents the protein expression level and Y- axis represents the proteins.

Six major proteins important for virus entry into cells replication such as ORF1ab, N, nsp9, S, nsp3 and H [1] were found expressed in the infected NOPS following high throughput proteomic analysis (Table 1). A total of 37 proteins of host humans were found differentially regulated on infection with SARS-CoV-2 (Table 2) (Fig 1). The differentially regulated proteins include 21 up regulated and one down regulated protein for having more than one log fold differential regulation (Table 2) (Fig 1). Proteins such as HIST1H3A, H2AFZ, HIST1H4A, S100A9, GSTA1, DPYSL5 and S100A8 were found to be up regulated by 4 log fold changes. ANXA2, BPIFA1, AXNA1, HIST1H2BK and AKR1C4 were up regulated by 3 log fold change. Proteins that were up regulated 1–3 fold were: LTF, ATP5F1A, YWHAQ, ANXA5, LYZ, PRDX1, DMBT1, LGAL53 and CAPS (Table 2) (Fig 1). Many of the up-regulated proteins are involved in host-defense functions and inflammatory reactions. The data generated from the proteomic analysis were submitted to PRIDE database and obtained PXD032150 project accession number (DOI: 10.6019/PXD032150).

**Table 1:** List of viral proteins which were found expressed in the COVID infected NOPS samples.

S.No	UniProt ID	Protein Symbol	Protein Name
1	YP_009724389.1	ORF1ab	ORF1ab polyprotein [Severe acute respiratory syndrome coronavirus 2]
2	YP_009724397.2	N	nucleocapsid phosphoprotein [Severe acute respiratory syndrome coronavirus 2]
3	YP_009742616.1	nsp9	nsp9 [Severe acute respiratory syndrome coronavirus 2]
4	YP_009724390.1	S	surface glycoprotein [Severe acute respiratory syndrome coronavirus 2]
5	YP_009742610.1	nsp3	nsp3 [Severe acute respiratory syndrome coronavirus 2]
6	YP_009725308.1	H	helicase [Severe acute respiratory syndrome coronavirus 2]

**Table 2:** List of proteins which were found differentially regulated in the infected NOPS samples against control negative samples.

S.No	UniProt ID	Protien Symbol	Protein Name	Score	Coverage	Fold change
1	P68431	HIST1H3A	Histone H3.1 OS = Homo sapiens OX = 9606 GN = HIST1H3A PE = 1 SV = 2 - [H31_HUMAN]	9.80	14.71	4.7
2	P0C0S5	H2AFZ	Histone H2A.Z OS = Homo sapiens OX = 9606 GN = H2AFZ PE = 1 SV = 2 - [H2AZ_HUMAN]	5.91	7.03	4.7
3	P62805	HIST1H4A	Histone H4 OS = Homo sapiens OX = 9606 GN = HIST1H4A PE = 1 SV = 2 - [H4_HUMAN]	20.94	41.75	4.6
4	P06702	S100A9	Protein S100-A9 OS = Homo sapiens OX = 9606 GN = S100A9 PE = 1 SV = 1 - [S10A9_HUMAN]	9.84	24.56	4.5
5	P08263	GSTA1	Glutathione S-transferase A1 OS = Homo sapiens OX = 9606 GN = GSTA1 PE = 1 SV = 3 - [GSTA1_HUMAN]	2.64	4.05	4.3
6	Q9BPU6	DPYSL5	Dihydropyrimidinase-related protein 5 OS = Homo sapiens OX = 9606 GN = DPYSL5 PE = 1 SV = 1 - [DPYL5_HUMAN]	2.38	1.24	4.2
7	P05109	S100A8	Protein S100-A8 OS = Homo sapiens OX = 9606 GN = S100A8 PE = 1 SV = 1 - [S10A8_HUMAN]	5.20	19.35	4.2
8	P07355	ANXA2	Annexin A2 OS = Homo sapiens OX = 9606 GN = ANXA2 PE = 1 SV = 2 - [ANXA2_HUMAN]	31.80	23.60	3.9
9	Q9NP55	BPIFA1	BPI fold-containing family A member 1 OS = Homo sapiens OX = 9606 GN = BPIFA1 PE = 1 SV = 1 - [BPIA1_HUMAN]	7.43	13.67	3.3
10	P04083	ANXA1	Annexin A1 OS = Homo sapiens OX = 9606 GN = ANXA1 PE = 1 SV = 2 - [ANXA1_HUMAN]	38.93	27.75	3.2
11	O60814	HIST1H2BK	Histone H2B type 1-K OS = Homo sapiens OX = 9606 GN = HIST1H2BK PE = 1 SV = 3 - [H2B1K_HUMAN]	2.96	8.73	3.1
12	P17516	AKR1C4	Aldo-keto reductase family 1 member C4 OS = Homo sapiens OX = 9606 GN = AKR1C4 PE = 1 SV = 3 - [AK1C4_HUMAN]	3.11	2.17	3.1
13	P02788	LTF	Lactotransferrin OS = Homo sapiens OX = 9606 GN = LTF PE = 1 SV = 6 - [TRFL_HUMAN]	2.54	1.13	2.7
14	P25705	ATP5F1A	ATP synthase subunit alpha, mitochondrial OS = Homo sapiens OX = 9606 GN = ATP5F1A PE = 1 SV = 1 - [ATPA_HUMAN]	2.73	1.81	2.5
15	P27348	YWHAQ	14-3-3 protein theta OS = Homo sapiens OX = 9606 GN = YWHAQ PE = 1 SV = 1 - [1433T_HUMAN]	2.77	3.67	2.5
16	P08758	ANXA5	Annexin A5 OS = Homo sapiens OX = 9606 GN = ANXA5 PE = 1 SV = 2 - [ANXA5_HUMAN]	6.08	8.44	2.4
17	P61626	LYZ	Lysozyme C OS = Homo sapiens OX = 9606 GN = LYZ PE = 1 SV = 1 - [LYSC_HUMAN]	2.45	8.11	2.3
18	Q06830	PRDX1	Peroxiredoxin-1 OS = Homo sapiens OX = 9606 GN = PRDX1 PE = 1 SV = 1 - [PRDX1_HUMAN]	3.58	6.03	1.8
19	Q9UGM3	DMBT1	Deleted in malignant brain tumors 1 protein OS = Homo sapiens OX = 9606 GN = DMBT1 PE = 1 SV = 2 - [DMBT1_HUMAN]	2.45	6.96	1.7
20	P17931	LGALS3	Galectin-3 OS = Homo sapiens OX = 9606 GN = LGALS3 PE = 1 SV = 5 - [LEG3_HUMAN]	3.26	4.40	1.5
21	Q13938	CAPS	Calcyphosin OS = Homo sapiens OX = 9606 GN = CAPS PE = 1 SV = 1 - [CAYP1_HUMAN]	2.49	5.29	1.4
22	P08246	ELANE	Neutrophil elastase OS = Homo sapiens OX = 9606 GN = ELANE PE = 1 SV = 1 - [ELNE_HUMAN]	3.23	3.75	0.9
23	Q13835	PKP1	Plakophilin-1 OS = Homo sapiens OX = 9606 GN = PKP1 PE = 1 SV = 2 - [PKP1_HUMAN]	3.15	3.88	0.7
24	P02452	COL1A1	Collagen alpha-1(I) chain OS = Homo sapiens OX = 9606 GN = COL1A1 PE = 1 SV = 5 - [CO1A1_HUMAN]	5.59	1.23	0.6
25	Q60I27	ALS2CL	ALS2 C-terminal-like protein OS = Homo sapiens OX = 9606 GN = ALS2CL PE = 1 SV = 1 - [AL2CL_HUMAN]	5.84	1.99	0.6
26	P08123	COL1A2	Collagen alpha-2(I) chain OS = Homo sapiens OX = 9606 GN = COL1A2 PE = 1 SV = 7 - [CO1A2_HUMAN]	2.55	0.66	0.3
27	Q6UXH9	PAMR1	Inactive serine protease PAMR1 OS = Homo sapiens OX = 9606 GN = PAMR1 PE = 1 SV = 1 - [PAMR1_HUMAN]	6.09	1.53	0.3
28	P25391	LAMA1	Laminin subunit alpha-1 OS = Homo sapiens OX = 9606 GN = LAMA1 PE = 1 SV = 2 - [LAMA1_HUMAN]	2.90	0.33	0.2
29	P78552	IL13RA1	Interleukin-13 receptor subunit alpha-1 OS = Homo sapiens OX = 9606 GN = IL13RA1 PE = 1 SV = 1 - [I13R1_HUMAN]	3.10	7.96	0.0
30	O95622	ADCY5	Adenylate cyclase type 5 OS = Homo sapiens OX = 9606 GN = ADCY5 PE = 1 SV = 3 - [ADCY5_HUMAN]	3.13	1.67	-0.3
31	O00398	P2RY10	Putative P2Y purinoceptor 10 OS = Homo sapiens OX = 9606 GN = P2RY10 PE = 2 SV = 1 - [P2Y10_HUMAN]	3.09	7.96	-0.4
32	Q96PX9	PLEKHG4B	Pleckstrin homology domain-containing family G member 4B OS = Homo sapiens OX = 9606 GN = PLEKHG4B PE = 1 SV = 4 - [PKH4B_HUMAN]	3.46	2.44	-0.5
33	Q8NF91	SYNE1	Nesprin-1 OS = Homo sapiens OX = 9606 GN = SYNE1 PE = 1 SV = 4 - [SYNE1_HUMAN]	3.13	0.43	-0.5
34	P50542	PEX5	Peroxisomal targeting signal 1 receptor OS = Homo sapiens OX = 9606 GN = PEX5 PE = 1 SV = 3 - [PEX5_HUMAN]	3.08	5.16	-0.6
35	P29508	SERPINB3	Serpin B3 OS = Homo sapiens OX = 9606 GN = SERPINB3 PE = 1 SV = 2 - [SPB3_HUMAN]	2.81	2.56	-0.7
36	Q01995	TAGLN	Transgelin OS = Homo sapiens OX = 9606 GN = TAGLN PE = 1 SV = 4 - [TAGL_HUMAN]	3.06	3.98	-0.9
37	Q6NWY9	PRPF40B	Pre-mRNA-processing factor 40 homolog B OS = Homo sapiens OX = 9606 GN = PRPF40B PE = 1 SV = 1 - [PR40B_HUMAN]	3.16	4.13	-1.6

### 2.2. Gene expression analysis

**Fig 2:**
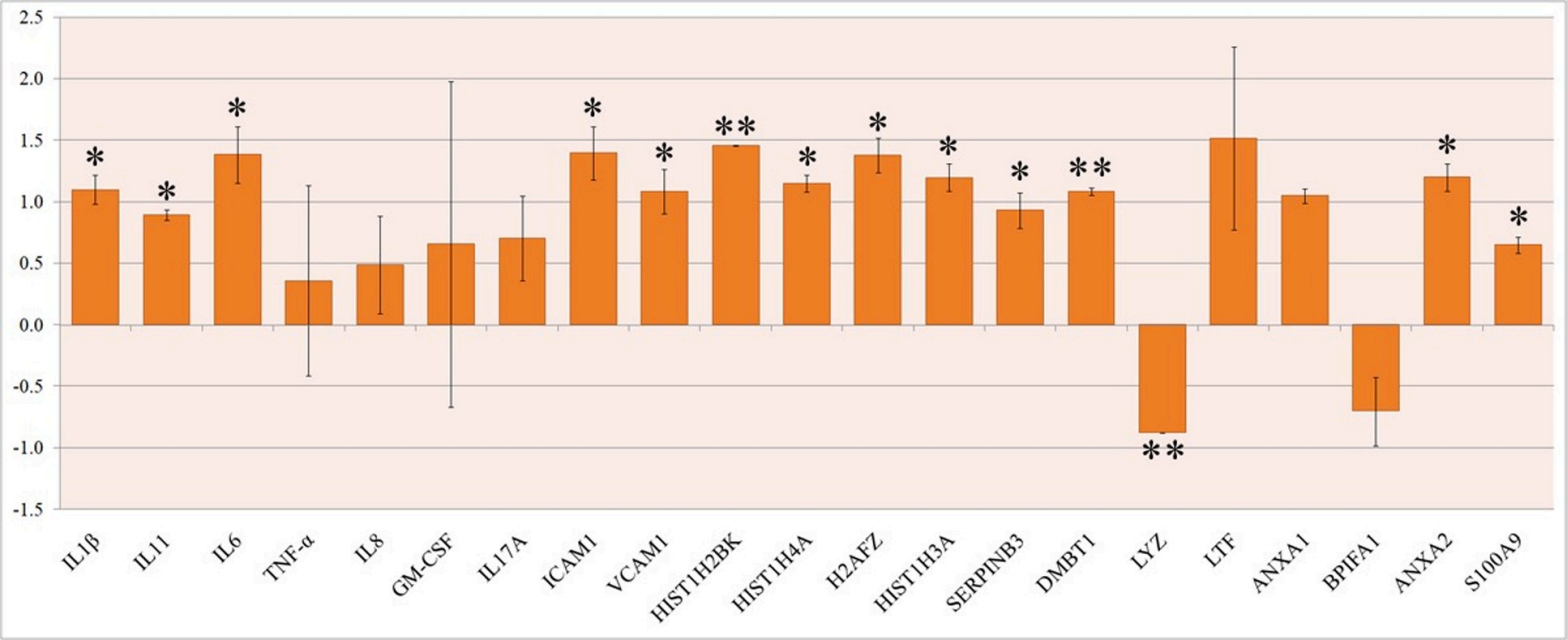
Expression analysis of genes. qRTPCR expression analysis of genes associated with infection. The bar represents the relative expression of the gene in SARS-CoV-2 infected samples against negative controls. The X-axis represents the genes and Y- axis represents the differential fold expression.

Genes of proteins involved in immune responses [16] and those that were observed to be up regulated in the proteomics analysis were analyzed for their expression involving RTPCR during SARS-CoV-2 infection using respective gene specific primers (Table 3). Based on RTPCR analysis it was found that majority of the selected genes were significantly up regulated on viral infection. IL1b, IL11, IL6, ICAM1, VCAM1, HIST1H2BK, HIST1H4A, H2AFZ, HIST1H3A, SERPINB3, DMBT1, ANXA1, ANXA2 were found up-regulated by one log fold change (Fig 2). LYZ is the only gene which is found down regulated upon SARS-CoV-2 infection (Fig 2). Proteins which were found up regulated were also up regulated at the gene level through RTPCR.

**Table 3:** List of genes and their primer pairs used for the RTPCR validation.

S. No	Gene	Symbol	Forward Primer	Reverse Primer
1	Interleukin 1β	IL1β	TAGATCCCAAAAATTACCCAAAGA	TGCATGGTGAAGTCAGTTATATCC
2	Interleukin 11	IL11	TGCACAGCTGAGGGACAAATTC	GTCTTCAGGGAAGAGCCACCTG
3	Interleukin 6	IL6	AATCTGGATTCAATGAGGAGACTT	GCATCTAGATTCTTTGCCTTTTTC
4	Tumor necrosis factor α	TNF-α	CAGTCAGATCATCTTCTCGAACC	CTTGAAGAGGACCTGGGAGTAGAT
5	Interleukin 8	IL8	AAGACATACTCCAAACCTTTCCAC	AATTCTCAGCCCTCTTCAAAAACT
6	Granulocyte-macrophage colony-stimulating factor	GM-CSF	GTCATCTCAGAAATGTTTGACCTC	TTCAAAGGTGATAATCTGGGTTG
7	Interleukin 17A	IL17A	ACCTCATTGGTGTCACTGCTACT	CGGTTGTAGTAATCTGAGGACCTT
8	Intercellular adhesion molecule 1	ICAM1	CGAGCTCAAGTGTCTAAAGGATG	GCTACCACAGTGATGATGACAATC
9	Vascular cell adhesion molecule 1	VCAM1	AGGAGACACTGTCATCATCTCTTG	TTGTGAGCCAACTTTGTTTTTAGA
10	Histone H2B type 1	HIST1H2BK	GAATCATGAACTCCTTCGTCAAC	TACTTAGCGCTGGTGTACTTGGT
11	Histone H4	HIST1H4A	AAGCGGATCTCTGGTCTGATCTAC	TTAACCGCCAAAGCCATAAAG
12	Histone H2A.Z	H2AFZ	CACCTAAAATCTAGGACGACCAGT	GAGAATCCAATTCTTCATCTCCAC
13	Histone H3.1	HIST1H3A	CTAAGCAAACTGCTCGGAAGTCTA	TTTACGAATAAGCAGTTCAGTGGA
14	Serpin B3	SERPINB3	GCAATACACATCTTTTCATTTTGC	CATATTCTGCAAACTTGTCCATTC
15	Deleted in malignant brain tumors 1	DMBT1	TTTTGACGTGAACATTTCCTTTTA	AAGACGTGAAGTCATTGGAGTATG
16	Lysozyme C	LYZ	AGAACTCTGAAAAGATTGGGAATG	GTTTTGCCATCATTACACCAGTAG
17	Lactotransferrin	LTF	CTATAGGGACACTTCGTCCATTCT	ACCAGAGTAGCTGAAGTACGGTTC
18	Annexin A1	ANXA1	TAAGGCCATAATGGTTAAAGGTGT	TTAGCAGAGCTAAAACAACCTCCT
19	BPI fold-containing family A member	BPIFA1	CTGGACATCACTGCAGAAATCTTA	ACTCAGGCAGGACTTTATTCAAGA
20	Annexin A2	ANXA2	TATGAACTGATTGACCAAGATGCT	TCTCCTTTAACCTCTTTCCTGATG
21	Protein S100	S100A9	CATAGAGACCATCATCAACACCTT	AGCTGCTTGTCTGCATTTGTGT
22	Glyceraldehyde 3-phosphate dehydrogenase	GAPDH	ATGACATCAAGAAGGTGGTGAAG	CTGTAGCCAAATTCGTTGTCATAC

### 2.3. Network pathway analysis

**Fig 3:**
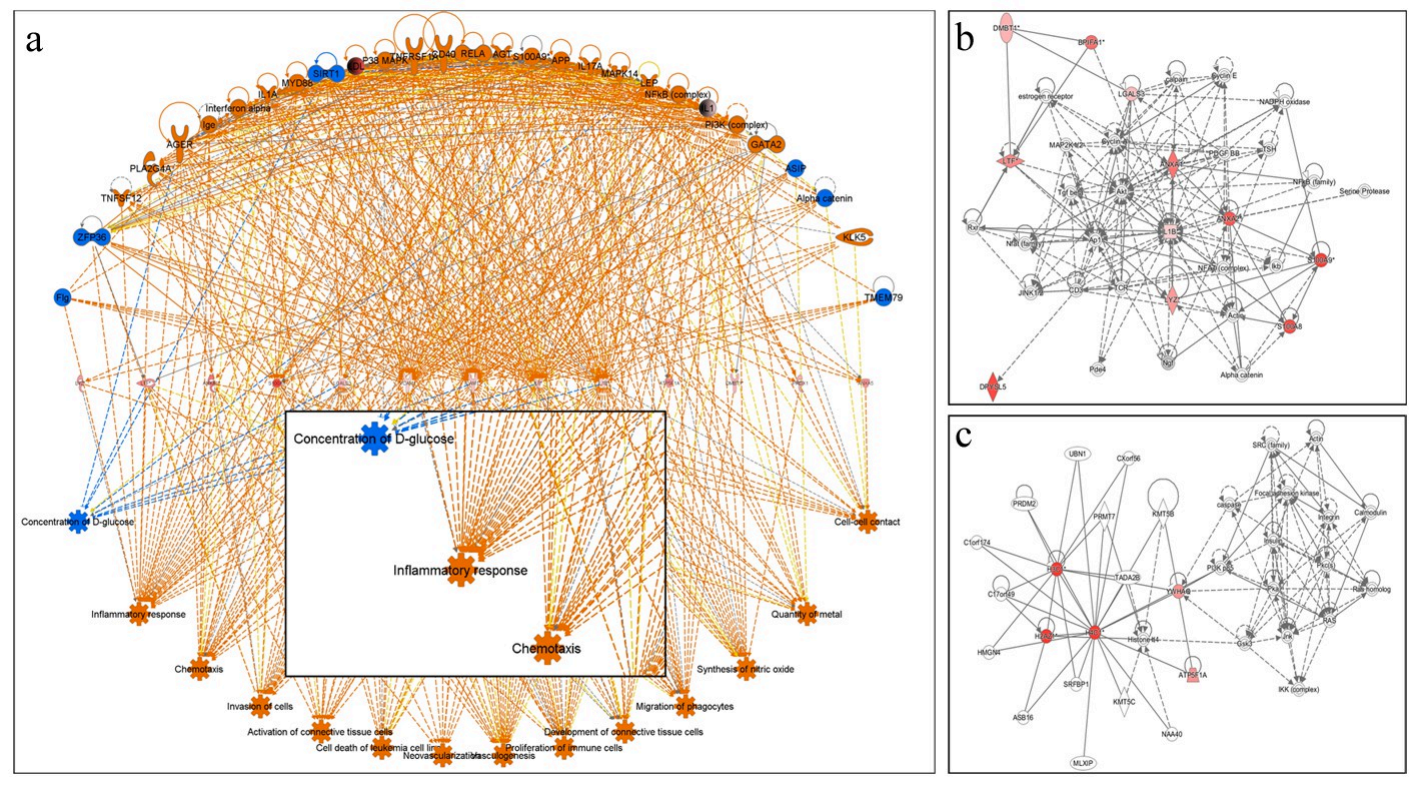
Network pathway analysis. Network pathway analysis of the genes/proteins associated with infection in NOPS samples. a. Interaction network based on IPA analysis. b and c. Two most significant network associated with infection.

**Fig 4:**
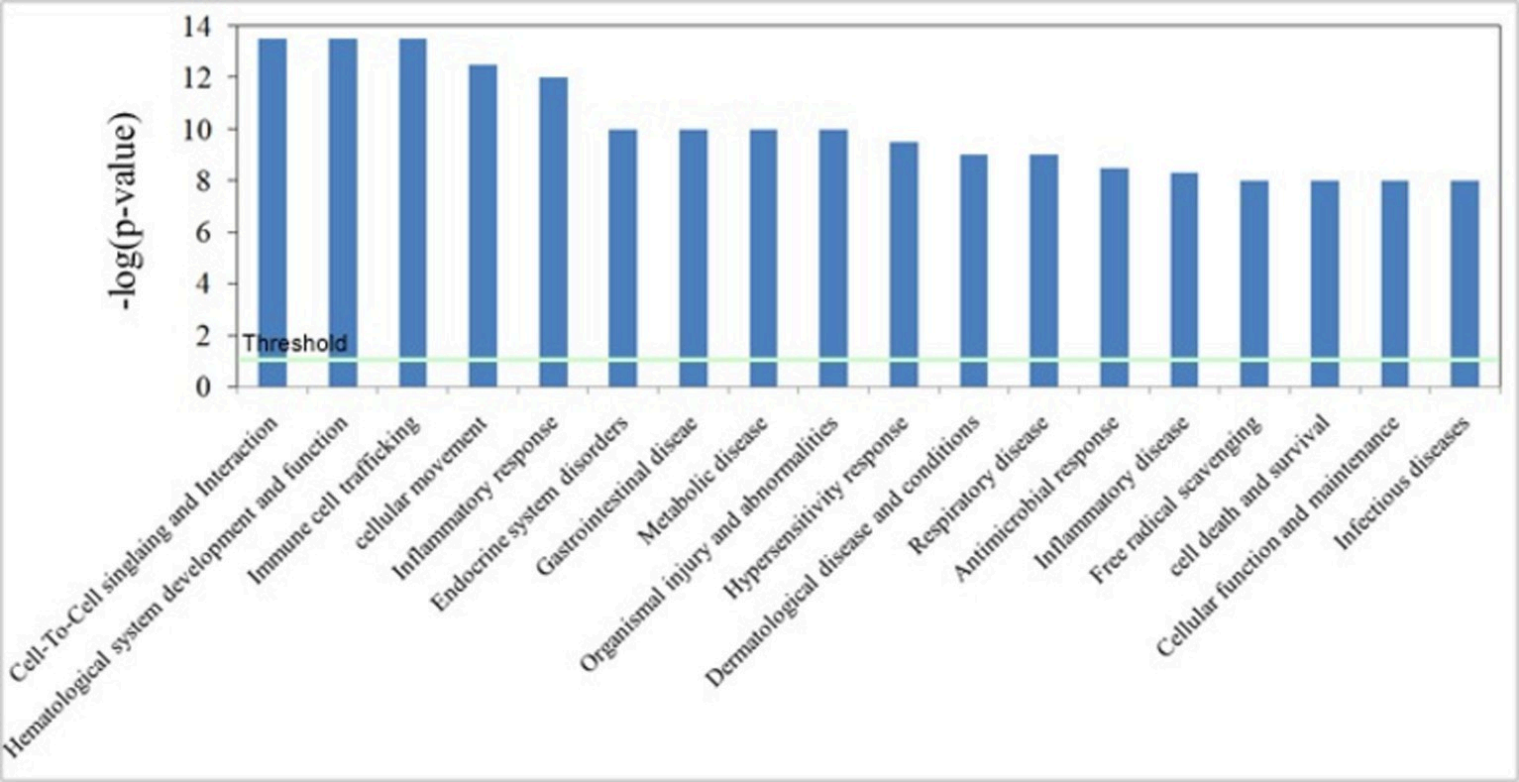
Functions of proteins or genes associated with infection. List of functions (or disease) associated with the differentially expression proteins/genes which are associated with infection.

Based on network pathway analysis it was found that inflammatory response network pathway was highly associated with the differentially regulated proteins and genes observed and selected in the study. Genes/proteins (16 in number) which were found differentially regulated in the NOPS samples for SARS-CoV-2 infection were found associated with inflammatory response pathway (Fig 3A). Cellular movement/cell to cell signalling and cellular assembly/organization were the two major canonical pathways found to be associated with the differentially regulated proteome of infected NOPS (Fig 3B). The major functions associated with the identified and differentially regulated proteome includes cell to cell signalling and interaction, immune cell trafficking, cellular movement, inflammatory response and hypersensitive response (Fig 4).

### 2.4. Cytokine array and western blot analysis

**Fig 5:**
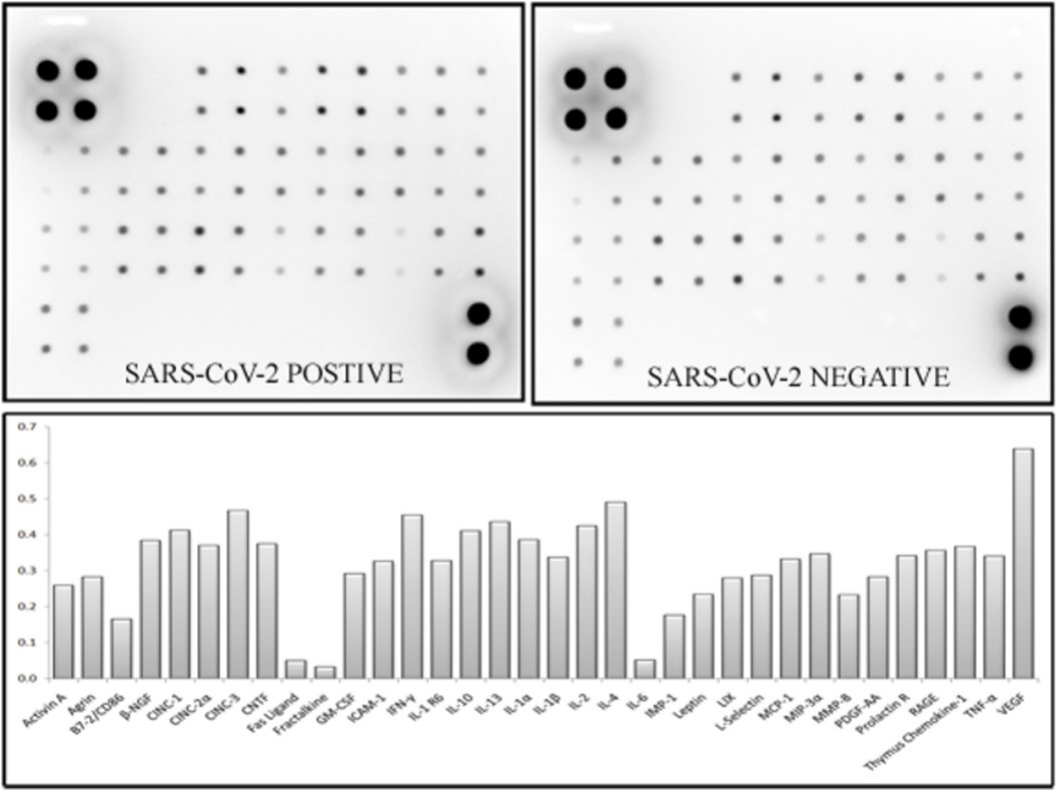
Cytokine protein array analysis. Cytokine protein array analysis for SARS-CoV-2 positive and negative samples. Bottom panel represent the relative expression of the protein based on cytokine protein array analysis.

**Fig 6:**
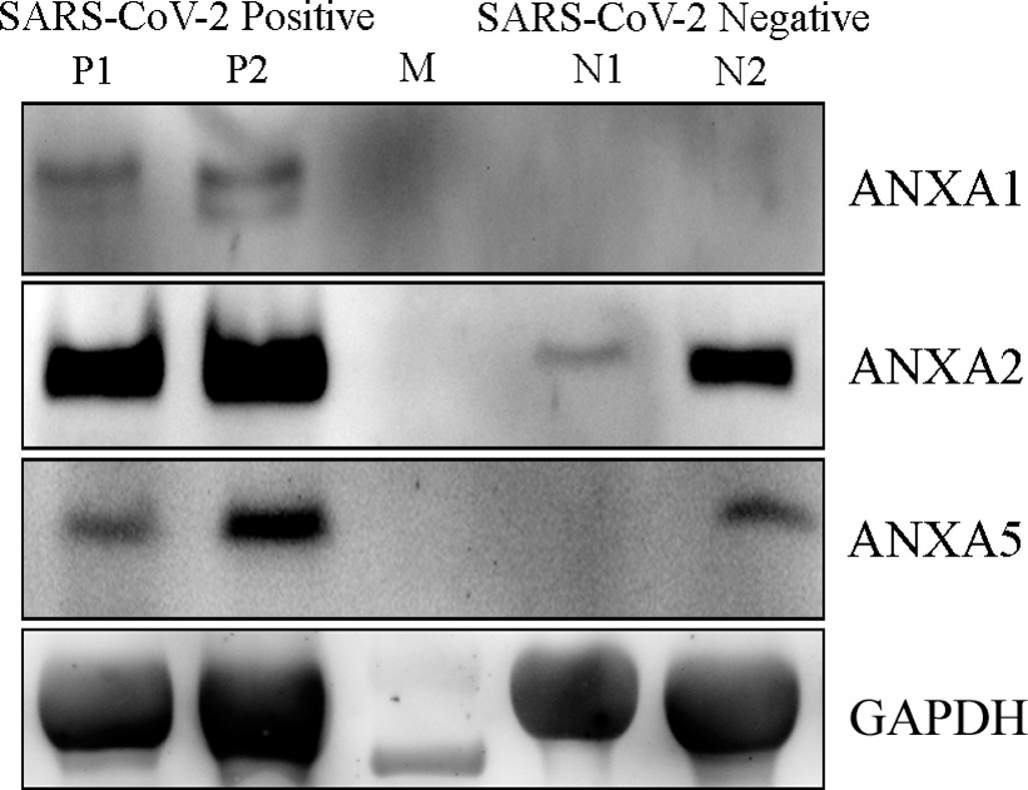
Analysis of Annexins. Western blot analysis for ANXA1, ANXA2 and ANXA5 proteins in infected and non-infected NOPS samples. P1 and P2 indicates the SARS-CoV-2 positive samples; M denotes the protein molecular weight standard and N1 and N2 denotes the SARS-CoV-2 negative samples.

Based on cytokine array analysis it was found that almost all the proteins represented for the cytokine pathway [16] were found to be up regulated in the infected Nasopharyngeal region samples against the control NOPS samples (Fig 5). Majority of the interleukins, VEGF, CNTF and CINC-2 were found to be majorly up regulated for COVID infection based on cytokine array analysis (Fig 5). Based on western blot analysis it was found that ANXA1, ANXA2 and ANXA5 were found to be up regulated by several fold in SARS-CoV-2 positive NOPS samples against control NOPS samples (Fig 6). ANXA2 was found up regulated majorly in the infected samples.

## 3. Discussion

The initial site of SARS-COV-2 infection is the upper airways. As the disease progresses, respiratory difficulties have been observed in severe cases [2]. The expression of two main SARS-COV-2 receptors, angiotensin converting enzyme-2 (ACE2) and transmembrane protease serine 2 (TMPRSS), was found to be high in the upper airway tract mucosa [11]. The nasopharyngeal region plays a major role in mucosal immunity against SARS-COV-2 infection by activating lymphocytes, B cells, and other cellular components, as well as activating antigen specific immunity [4]. The oral cavity was also reported to be implicated in various immunological responses to SARS-COV-2 infection [11]. In fact, the test for viral infection is from swabs from this region which are subjected to RTPCR. The swabs would contain epithelial and immune cells [15, 17]. We have shown that when cells from this region were analyzed for the expression of host-defense peptides-the defensins, they were significantly down-regulated [12]. In this paper, we described the effect of SARS-CoV-2 infection in patients on the expression of cytokine genes and proteomics profile of cells from the nasopharyngeal region using high throughput comparative proteomic analysis.

We have analyzed pooled samples from individuals who were tested COVID19 positive by RTPCR test and individuals who were COVID19 negative. Samples were obtained from individuals during early days of the pandemic in 2020. The major viral proteins were detected in the infected nasopharyngeal samples. They were SARS-CoV-2 non-structural proteins that are responsible for viral transcription, replication, proteolytic processing, suppression of host immune responses and suppression of host gene expression (Table 1). The nucleocapsid protein is an RNA-binding protein that is essential for viral assembly into a ribonucleoprotein complex and also functions in viral budding.

The host proteins found to be differentially regulated for SARS-CoV-2 infections are involved in various aspects of immune response, particularly neutrophil activation. Our proteomic profile shows that HIST1H3A, H2AFZ, HIST1H4A, GSTA1, DPYSL5, S100A9, and S100A8 proteins were found to be up regulated by more than 4 log fold changes. Several neutrophil activated proteins and DNA binding protein such as Histones have a significant role in inflammation (Figs 1 and 2). Histones are prominent component of neutrophil extracellular traps (NETs), which causes inflammation and thrombosis [18]. Neutrophil activated proteins S100A8/A9 or calprotectin [19], DPYSL5 [20], and neutrophil elastase (ELANE) expression were all linked to COVID-19 infection and severity through the formation of NETs, which causes acute lung injury [21]. It is noteworthy to explore anti-neutrophil therapy and suggest that it could be a potential therapeutic target for viral infections.

In SARS-COV-2 infection, reactive oxygen species (ROS) cause oxidative stress in host cells and stimulate the production of cytokines, antioxidants, transcription factors, dendritic cells, and macrophages [22]. Our findings (Table 2) show the detoxifying enzyme Glutathione S transferase (GSTA1) [22], antioxidant Peroxiredoxin-1 (PRDX1) [22], and antibacterial enzyme Lysozyme C (LYZ) [4, 23] protein upregulation during SARS-COV-2 infection, indicating a significant oxidative defense mechanism. The severity of COVID-19 disease has been linked to elevated levels of ROS production [24]. As a result, our study strongly suggests that further research on ROS-mediated markers will help in identifying infection severity at an early stage.

Several other proteins as described in Table 2 are also associated with inflammatory responses and apoptosis. It is interesting to see the up regulation of Annexins (Figs 2 and 6) (Table 2). Annexin family proteins are calcium dependent phospholipid binding proteins. Annexin A1 (ANXA1), Annexin A2 (ANXA2), Annexin A5 (ANXA5) are predominantly found in SARS-CoV-2 infected host cells and are involved in the pro-inflammatory response and thrombosis [25, 26]. Recent reports indicate that auto-antibodies to Annexin 2 is detected in COVID19 patients, suggesting that it plays an important role in pathophysiology [14, 26].

Our proteomic findings are also consistent with *in vitro* studies that showed SARS-CoV-2 infection activated host immune proteins. Cytokines are a major component of the innate immune response and are produced by lymphocytes and granulocytes [27–29]. During inflammatory reaction, Host cells pathogen-recognition receptors (PRRs) triggers cytokines in host system [27–30]. Cytokines are divided into two types: pro-inflammatory cytokines, which release excessive cytokines in response to infection, and anti-inflammatory cytokines, which regulate pro-inflammatory cytokines [27–29]. Pro-inflammatory cytokines such as IL-1, IL-10, IL-6, and TNF- α were found in significant concentrations in COVID-19 individuals, causing a cytokine storm during SARS-CoV-2 infection, which has been linked to ARDS, multi-organ failure, and increased candidate risk [28, 31]. Tumor necrosis factor α (TNF-α), regulates leukocyte trafficking by stimulating cell adhesion molecules such ICAM-1, VCAM-1, and selectins and it has been linked to a wide range of immunological disorders [32]. Our study found that pro-inflammatory cytokines such as IL-1, IL-6, IL-10, and TNF- α, as well as other cytokines proteins were upregulated (Fig 5). At genomic expression, IL-6 found to be significantly up regulated with more than 1 log fold change (Fig 2). Despite the lack of disease severity data, our findings are consistent with previous research [4] and suggest that IL-6 can be used as a marker for COVID-19 disease severity. Some proteins, such as Deleted in malignant brain tumours 1 (DMBT1) and Lactotransferrin (LTF), bind to growth factors and inhibit IL-6 production [33, 34], suggesting that they could be antiviral. Our study indicates that a preliminary screen of host cells using global proteomics provides a detailed view of viral effects and leads to potential therapeutics.

Although infection by corona virus was manifested in a mild form earlier on [1, 2], the pandemic due to the corona virus SARS-CoV-2 that started in 2019 had a devastating effect on health services globally. Despite extensive efforts, there is no drug specifically to treat COVID19. There have been extensive researches on various aspects on the virology of SARS-CoV-2, not only to understand the disease, but also to devise effective treatment. Development of vaccines at warp speed has been effective in controlling the disease [35], but data on long term protection are not available. The symptoms are highly variable among infected individuals and also appear to depend on the infecting strain. Responses by various populations may differ considerably. Hence, it is important to investigate protein and gene expression from patients from different geographical locations and ethnicity. It is important to examine various biochemical parameters on the onset of infection particularly from the initial site of infection and during early days of the pandemic, to understand various aspects of the disease. By a combination of proteomics analysis, gene expression studies as well as in the blot array, we were able to detect several proteins related to inflammation and auto immunity such as histones and annexins and up regulated cytokines. Clearly a multi-dimensional approach is necessary to obtain a complete picture on SARS-CoV-2 infection. Such an approach would facilitate therapeutic interventions.

## 4. Methods

### 4.1. Sample collection

Human nasopharyngeal/oropharyngeal swab samples were collected from CSIR—Centre for Cellular and Molecular Biology (CCMB) COVID19 diagnostic facility archive in viral transport media (VTM) [12]. A total of 80 VTM swab samples (collected from Government hospital, Hyderabad, Telangana, India during early phase of COVID infection in 2020) were selected for genomics analysis. A further 12 VTM swab samples were selected for quantitative differential proteomics analysis. The samples were inactivated in their respective lysis buffer. The study was executed as per the approval of CCMB Institutional biosafety and institutional ethical approval (82/2020).

### 4.2. Protein extraction and iTRAQ labelling

A total of 12 VTM swab samples were grouped into 2 with each group consisting of 3 positive and 3 negative SARS-CoV-2 VTM samples were taken for the quantitative differential proteomics study. Total protein was extracted from the pooled SARS-CoV-2 VTM samples at the CCMB BSL3 lab facility. The pooled samples were centrifuged at high RPM, and the resulting pellet was washed with 1% PBS, resuspended in protein solubilization buffer, (7 M urea, 2 M thiourea, 18 mM Tris–HCl, 4% CHAPS, 14 mM Trizmabase, Triton X 0.2%, 50 mM DTT and EDTA free protease Inhibitor cocktail mix) and sonicated for 10 minutes. After brief centrifugation, the supernatant was collected and quantified using the amido black method [36]. A total of 200ug of protein was in-gel trypsin digested and labelled with iTRAQ label as per previously mentioned protocol [36–40]. SARS-COV-2 negative samples were labelled with iTRAQ 114 and 115, and positive samples were labelled with 116 and 117. All the labelled peptides were pooled and analysed using Liquid Chromatography Mass Spectrometry (LCMS/MSMS) in the Q-Exactive HF mass spectrometer coupled to EASY-nLC 1200 system. Peptides were eluted through PepMap^TM^ RSLC C18 column (Thermo Scientific) with 150 min gradient. The electro spray voltage was set to 2.2 KV and the capillary temperature was set to 310°C. The mass spectrometers were used in a Top 10 Data Dependent DD-MS2 and selected ion monitoring data dependent acquisition ddSIM. The raw data were analyzed using Sequest HT proteome discoverer 1.4 (Thermo Scientific), with 1% FDR percolator and XCorr (Score Vs Charge). The parameters are summarized in Supplementary Table 1. The obtained data were analyzed against the human proteome and SARS-CoV-2 proteome database separately. The identified SARS-COV-2 and host proteins were listed. Differential expression analysis was carried for the obtained host proteins against control SARS-CoV-2 negative samples. The proteomic data obtained from the study was submitted to PRIDE database.

### 4.3. Real-time PCR analysis

Total RNA was extracted from the selected individual VTM swab samples using KingFisherTM Flex System (Thermo Fisher Scientific Inc., USA). A total of 80 VTM RNA samples were quantified and grouped into 8 with each group consisting 100ng of 5 positive and 5 negative samples. cDNA was synthesized (BioRad, USA) from 200 ng of each VTM group. Real-time PCR analysis was performed for the selected list of the gene using Applied BiosystemsViiA™ 7 Real-Time PCR System (USA) with gene-specific primers (Table 3). All primers were synthesized using Primer3 software and, the GAPDH gene was used for the data normalization. TB Green Premix Ex Taq 11 (TliRNaseH Plus) kit (Takara, Japan) with respective qRTPCR conditions (Annealing Tc-55°C or 60°C) and melt curve analysis were followed as previously mentioned protocol [12]. Differential expression analysis was performed using the obtained cycle threshold value.

### 4.4. Cytokine array and western blot analysis

Cytokine expression analysis was performed using Cytokine Array—RAT Cytokine antibody array (Abcam, USA) kit. A total of 300 μg proteins from two groups consisting of 6 positive and 6 negative samples were immunoblotted in the kit as per the manufacturer’s protocol. The obtained spot patterns were densitometrically analyzed using ImageJ software to estimate the expression level of cytokines in SARS-CoV-2 infection.

Protein expression analysis of ANXA1, ANXA2 and ANXA5 protein were analyzed involving western blot analysis. 25 μg of total protein electrophoresed in 10% SDS-PAGE and, immune blotted against respective antibodies [41]. GAPDH was used as a housekeeping protein.

### 4.5. Pathway analysis

The proteins and genes selected for the study were analyzed for network and pathway analysis using Ingenuity Pathway Analysis software-based. The network pathway, regulator effect and disease and function associated with the genes/protein were mapped.

## Supporting information

S1 Table Acquisition parameters used for LC-MS/MS analysis. (DOCX)
