# Supplementary material for: A proteomic perspective and involvement of cytokines in SARS-CoV-2 infection
Source: PLoS One. 2023 Jan 6;18(1):e0279998. doi: 10.1371/journal.pone.0279998 (PMC9821788; doi:10.1371/journal.pone.0279998)
Supplement: S1 Table — (DOCX) [file pone.0279998.s001.docx]

**Supplementary Table 1: Acquisition parameters used for LC-MS/MS analysis**

| **FULL MS / DD-MS² (TOPN)** | |
| --- | --- |
| General Runtime | 0 to 150 min |
| Polarity | positive |
| In-source CID | 0.0 eV |
| Default charge state | 2 |
| Inclusion | ? |
| Exclusion | ? |
| Tags | ? |
| Full MS |  |
| Microscans | 1 |
| Resolution | 60,000 |
| AGC target | 3e6 |
| Maximum IT | 100 ms |
| Number of scan ranges | 1 |
| Scan range | 400 to 1650 m/z |
| Spectrum data type | Profile |
| **dd-MS² / dd-SIM** | |
| Microscans | 1 |
| Resolution | 15,000 |
| AGC target | 1e5 |
| Maximum IT | 100 ms |
| Loop count | 10 |
| MSX count | 1 |
| TopN | 10 |
| Isolation window | 1.4 m/z |
| Isolation offset | 0.0 m/z |
| Scan range | 200 to 2000 m/z |
| Fixed first mass | 50.0 m/z |
| (N)CE / stepped (N)CE nce: | 30 |
| Spectrum data type | Profile |
| **dd Settings** | |
| Minimum AGC target | 1.00e3 |
| Intensity threshold | 1.0e4 |
| Apex trigger | ? |
| Charge exclusion | unassigned, 1, 6 - 8, >8 |
| Multiple charge states | all |
| Peptide match | preferred |
| Exclude isotopes | on |
| Dynamic exclusion | 15.0 s |
| If idle .. | do not pick others |
